# Knowledge, practice and communication barriers for oncology doctors in Chile when addressing the sexuality of their patients

**DOI:** 10.3332/ecancer.2024.1665

**Published:** 2024-02-06

**Authors:** Daniela Paz Rojas Miranda, V Constanza Micolich, Karen Goset, Sofia P Salas

**Affiliations:** 1 Postgraduate Unit, Faculty of Psychology, Diego Portales University, Santiago 8370076, Chile; 2 Carlos Van Buren Palliative Care Hospital, Valparaiso 2340000, Chile; 3 Bioethics Centre, Faculty of Medicine, Clinica Alemana Universidad del Desarrollo, Santiago 7610507, Chile

**Keywords:** clinical ethics, sexuality, quality of health care, communication barriers, cancer

## Abstract

**Introduction:**

Communication in a doctor–patient relationship constitutes a crucial aspect in medicine, and its multiple dimensions encompass a wide variety of ethical issues. Communication is particularly relevant in oncology, because it requires continually dealing with sensitive topics in one of the most highly vulnerable situations as a human: illness and proximity to death. Sexuality is one of these topics because it constitutes an area that is frequently affected by cancer and cancer treatment, which may include causing significant distress, the reinforcement of a negative self-image, relationship conflicts and a permanent memory of having cancer. The objective of this research is to describe the perception of knowledge and communications practices used by oncology doctors with respect to sexual health in the care of their patients, as well as the barriers found when it comes to confronting the topic.

**Methods:**

An exploratory quantitative, descriptive and cross-sectional study was carried out, in which a self-administered questionnaire was given to oncology doctors who practise in Chile. This questionnaire had 41 closed questions with answers on a Likert scale and was previously validated by being reviewed and applied to a pilot group of five professionals (one medical bioethics expert and four doctors in the field of oncology). The data were analysed with the SPSS statistical program v. 20, using descriptive statistics.

**Results:**

The main results show that the surveyed doctors consider sexuality to be an important part of patients’ quality of life. However, this finding does not align with the practices given for including it as part of clinical care. The professionals refer as the main barriers those that are attributed to the structural functioning of the institution, giving little value to those barriers related to personal aspects or those associated with patient characteristics and/or behaviors.

**Conclusion:**

The results of this study show that, despite oncology doctors seeing sexuality as an important aspect of the quality of life of their patients, they do not include the topic in clinical care. Given that one of the main barriers is obstacles relating to the institution, it is necessary to create political institutions that create the conditions for including this area as a relevant part of cancer patient care.

## Introduction

According to data provided by the World Health Organisation (WHO), cancer is the main cause of death worldwide. It is estimated that, in the year 2020, there were 20 million new cases of cancer and around 10 million deaths from the disease [1]. Similarly, a recent report from GLOBOCAN, with data obtained from 185 countries, estimates that the approximate overall risk of suffering from cancer over a lifespan is 25%, with significant differences based on the level of human development [2]. In Chile, the most recent data indicated that, in 2020, there were 54,227 new cancer diagnoses and 28,584 people died in the same period [3].

With growing health challenges, cancer and its treatment continue to have a significant multidimensional impact on people diagnosed with the condition and their friends and family. It significantly affects their quality of life, which requires an integral approach centred on the patient as a person. One of the most significant areas impacting the quality of life of cancer patients is sexuality [4, 5]. The WHO has defined sexuality as a central aspect of being a human, which includes sex, identity and gender roles, sexual orientation, eroticism, pleasure, intimacy and reproduction [6]. In this sense, sexuality is typically seen as being complicated, as much because of the physical and psychological aspects associated with cancer as the sociocultural factors that surround this condition [7].

Although there is a consensus on the importance of sexual health and the quality of life of people with cancer [8–10], the topic is scarcely covered in scientific literature or in clinical practice. Patients mention that the topic is practically absent in education on the condition, and that they would like the practitioners treating them to bring up these topics and offer them appropriate advice [11–17]. Research reveals the main barriers perceived by the health teams in order to address sexuality are related to challenges associated with the running of health institutions and ideas and preconceptions of healthcare professionals themselves in relation to the topic and to cancer [12, 18–21].

In Latin America there are few studies focused on the knowledge and practices of healthcare professionals when discussing sexuality in a clinical setting [22]. In the face of this lack of information, we consider it relevant to discover the knowledge, experience and communication barriers that oncology doctors from Chile have identified when approaching sexuality in the clinical care of their patients.

## Methods

### Design and materials

An exploratory, descriptive, quantitative, cross-sectional study was carried out using a self-directed questionnaire directed at oncology doctors who practise in Chile [23].

While the original Spanish text used masculine pronouns to discuss the healthcare professionals for practical linguistic reasons, the translated version uses the pronouns ‘they/them’ to indicate one or more individuals of unspecified gender.

The questionnaire was made up of 41 closed questions using a Likert scale with 5 possible answers, and was created on the digital platform SurveyMonkey. The questions were constructed based on a review of international studies, with similar objectives and surveys. [19, 24]. The survey took into account sociodemographic and labour-related variables, as well as questions to evaluate the level of self-assessed knowledge on sexuality in the context of illness, common communication practices related to the sexuality of their patients and barriers identified for including sexual health in a clinical setting [25, 26]. Prior to being used, the questionnaire was reviewed and given to a pilot group of five professionals (one doctor with expertise in bioethics and four oncologists), who made several suggestions regarding the wording of some questions. Their corrections were included in the final survey.

It is important to note that a standardised survey was not used, as our review indicated that there are no validated scales covering these issues in the Latin American population.

### Study population

The sampling was non-probabilistic; therefore, no statistical inferences were sought. For the sake of convenience, representatives from the Chilean Society of Radiotherapy [Sociedad Chilena de Radioterapia] and the Chilean Society of Medical Oncology [Sociedad Chilena de Oncología Médica] were contacted, and they distributed the survey amongst their members. Later, when the number of survey responses stalled, chain sampling was used (the ‘snowball effect’), to identify key participants, who were asked to pass on the information among their contacts [23]. The study was open from 28 September 2018 until 30 November 2018.

### Data analysis

To complete the analysis, the database was imported from SurveyMonkey, and subsequently refined to include only cases where the questionnaire was completed in its entirety. The data were analysed using the SPSS statistical package v. 20, using descriptive statistics. Due to the relatively small sample size, comparisons by sex, specialty or years in the profession were not performed, nor were conclusions drawn based on statistical significance. The purpose was to outline the distribution of practitioners’ perceptions within the areas covered by survey questions. In order to facilitate analysis of the results, the responses ‘agree’ and ‘strongly agree’ were grouped together, as were ‘disagree’ and ‘strongly disagree’.

## Results

### Demographic characteristics of individuals polled

A total of 84 valid questionnaires were obtained. Taking into account that a total of 200 practitioners were affiliated with the 2 scientific societies at the time of the study, the percentage of responses obtained was 42%. It should be noted that the distribution of sexes among those interviewed matched the distribution of registered doctors in the country (61.7% male) [27], as well as the proportion of men in the two societies (70% in oncology and 59% in radiotherapy). The median age was 44 (ages ranged from 29 to 74). Forty-four percent of the practitioners were employed in the private sector, 30% in the public and private sectors and 22% solely in the public sector (4% did not respond); 54% were radiotherapists and 46% were oncologists. Regarding professional experience, 30% had less than 6 years; 27% had 6–15 years, and 37% had over 15 years of experience (6% did not respond). To simplify the presentation of the results, the word ‘oncologists’ is intended to include both specialties.

### Self-assessed knowledge

**Figure 1.**
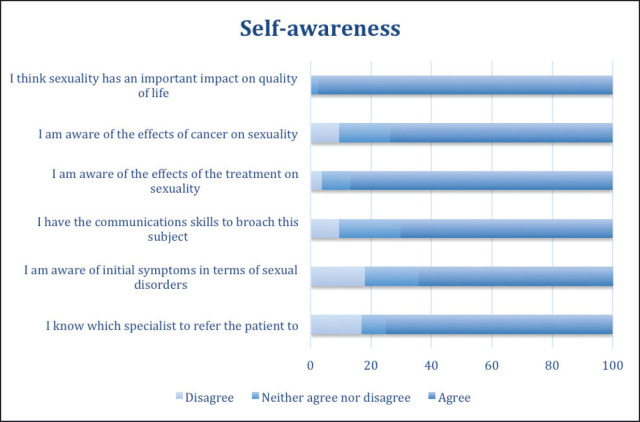
Oncologists' self-assessed knowledge regarding addressing sexuality in patient care.

As shown in Figure 1, 97.6% of the respondents considered sexuality to be important to their patients’ quality of life, and they report knowing how cancer affects their patients’ sexuality (73.8% responded ‘agree’); only 9.5% indicated that they were in disagreement with this statement. Likewise, the majority (86.9%) report knowing about the effect that cancer treatments have on patients’ sexual health. A high percentage of respondents (70.2%) believed that they had the necessary communication skills to address this issue; however, when they were asked if they had the knowledge to recognise initial signs of sexual disorders or whether they knew to which specialty to refer, the percentage of those responding ‘disagree’ increased (17.9% and 16.7%, respectively).

### Organisational barriers to clinical approaches to sexuality

**Figure 2.**
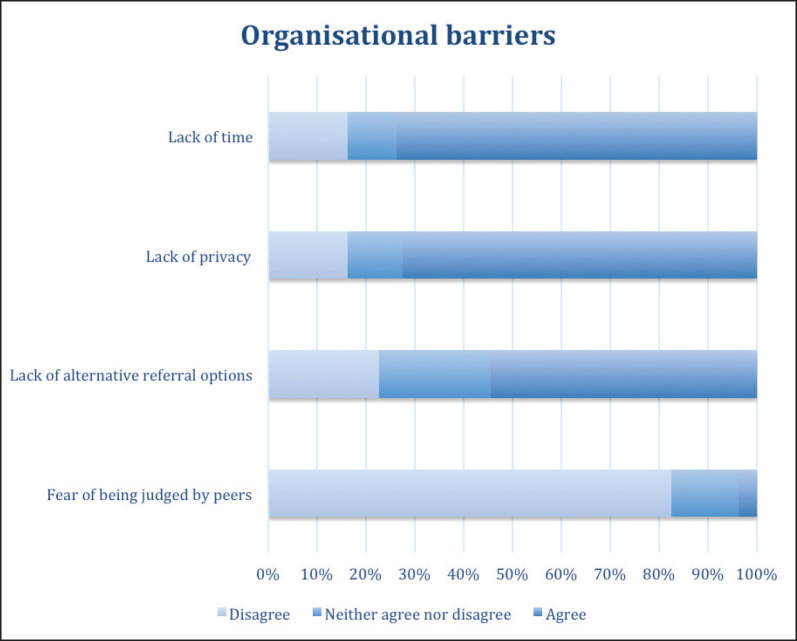
Doctors' perceived organisational barriers to addressing sexuality in patient care.

Regarding the difficulties that can present obstacles in addressing patients’ sexuality, participants cited the most relevant issues as: lack of time (73.8%), lack of privacy (72.5%), and, in third place, lack of alternatives for referral (54.5%). The majority (82.6%) considered that fear of judgment by their peers was not a relevant barrier to addressing this subject (Figure 2).

### Patient-related barriers

**Figure 3.**
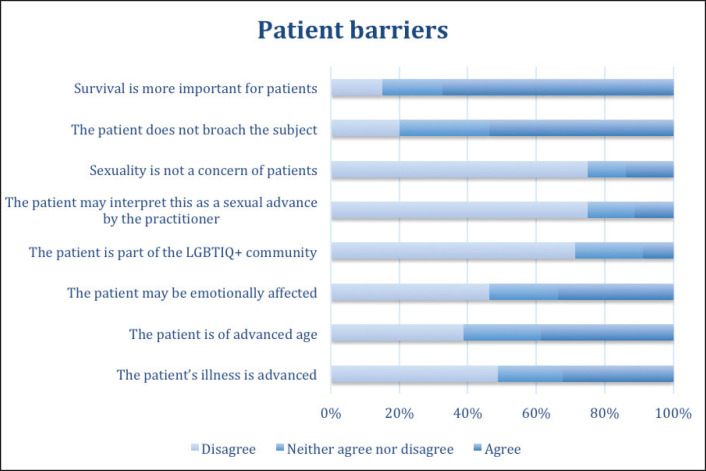
Patient characteristics that doctors consider to be barriers to addressing sexuality in patient care.

Regarding the patients’ difficulties that can present obstacles in addressing sexual health, the majority of practitioners thought that survival was more important for their patients than the issue of sexuality (67.5% of responses agreed), and that, because the patient did not bring up the issue, it was not appropriate to address it (53.8% agreed). On the other hand, 75% disagreed that sexuality was not a concern for their patients, and that addressing the topic could be interpreted as a sexual advance by the practitioner (75% disagreed). Disagreement was expressed with the statement that there was a barrier to addressing the topic if the patient was gay, lesbian or transgender (71.3% disagreed). In contrast, when asked about the following possible barriers – if the patient is emotionally affected by asking about these intimate topics, if the patient is elderly, or if the patient is in an advanced stage of disease – there were found to be nearly as many responses for ‘agree’ as for ‘disagree’ (Figure 3).

### Practitioner-related barriers

**Figure 4.**
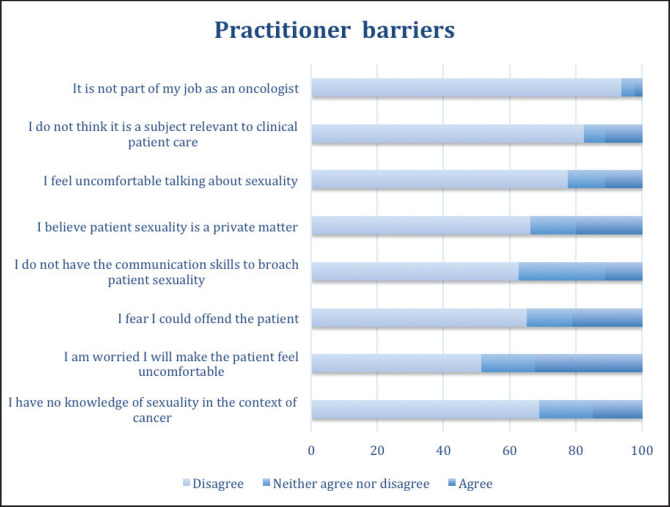
Practitioner-related barriers to addressing sexuality in clinical care.

Figure 4 shows that many of the possible barriers to addressing the topic of sexuality with their patients are not considered obstacles by the respondents. For example, 93.8% disagreed with the statement that sexual health is not part of their work as an oncologist, and the respondents also rejected the possible barriers that this was not a relevant topic in clinical care (82.5%) or that it was something private for patients (66.3%). Further, they disagree that the topic makes them feel uncomfortable (77.6%) and reject the statement that they do not possess communication skill to address it (62.6%) or that they lack sufficient knowledge (68.8%). It should be noted that one-third of the participants expressed concern about making their patient feel uncomfortable by bringing up the topic of sexuality (32.6% agreed).

### Communication practices

**Figure 5.**
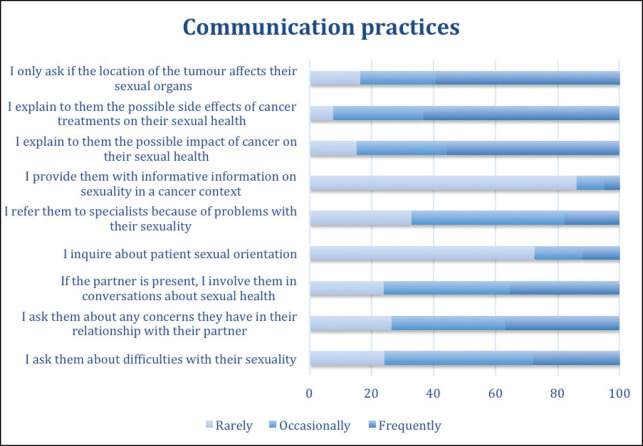
Communication practices of doctors when addressing sexuality in clinical care.

When asked about communication practices that they performed most frequently, the most relevant was that they tended to ask their patients about their sexuality only when the anatomic position of the lesion affected the sexual organs (59.5% answered ‘frequently’). The majority report informing patients about the possible side effects of cancer treatments on their sexual health (63.3%) and of the possible impact of cancer on their sexual health (55.7%). A majority (86.1%) also indicated that they rarely provide their patients with information about these aspects and only 12.7% enquire about the patients’ sexual orientation. 49.4% of the practitioners indicate that they only occasionally refer the patient to other specialists due to changes in sexuality and a third admit that they do it infrequently (32.9%). Likewise, they only occasionally (48.1%) ask their patients about difficulties with their sexuality or involve partners in conversations related to this topic (40.5%) (Figure 5).

## Discussion

According to our knowledge, this is the first study that addresses the perceptions of oncology doctors in Chile regarding their knowledge, practices and reported communication barriers when addressing discussions of sexuality with their oncology patients.

Its results show that participants recognise that sexuality is a central aspect of quality of life, as shown in international research data [20]. Additionally, they consider that they are able to handle how cancer and its treatments affect the sexual health of their patients in an optimal manner. This contrasts with international research, where the main reasons given by practitioners for not including sexual health in clinical care is a lack of training and knowledge in this area [19, 21, 24].

Aspects related to the functioning of the institution in which they were employed were identified as relevant barriers in addressing this topic. These included a lack of time and privacy and not having referral alternatives, vastly surpassing the variables related to the patient’s characteristics or the practitioner’s own barriers, which differs from international research. In a UK study, the main barriers identified were lack of training (79%), lack of time (67%) and embarrassment (50%) [28]. This study also identified age (61%), physical well-being (54%), sex (52%), marital status or whether or not they had a stable relationship (42%) as barriers that had an effect on approaching the topic of sexual health [28]. Similarly, in research carried out with Canadian and African practitioners, the majority considered that sexuality was not a priority for individuals with cancer, hence it also did not constitute a priority for physicians [29]. Another study with healthcare professionals from an oncological hospital in Saudi Arabia identified the lack of time, fear of being criticised by peers and the difficulty in finding a suitable space to talk about sexual problems as barriers [30].

These results differ from ours, where the most common barriers are identified as believing that survival is most important to the patient and that the patients do not address the issue, which contributes to the doctor considering that sexuality would not be a priority issue to include in the clinical discussion. These findings allow us to assume that, in clinical care with lower levels of time and intimacy (the main barriers identified in our study), sexuality begins to ‘compete’ with clinical objectives of another order. A third of our respondents believe that the presence of advanced cancer is an obstacle to addressing sexuality with their patient, which reflects the beliefs and/or difficulties that exist when talking about sexuality in the context of the end-of-life process, an aspect described in the literature as one of the most influential factors [4]. Socio-cultural meanings of sexuality, generally linked to youth, beauty and health, contrast with the characteristics associated with advanced cancer and even more so with the end-of-life process. Resistance to addressing sexuality in advanced stages has also been linked to discomfort among practitioners – such as the fear of being intrusive, lack of knowledge about the experience of sexuality at this stage of the disease and the lack of privacy as discussed earlier – can be even more problematic in the context of advanced illness. Even though interest in coital sexual relations has been seen to decrease as the pathology advances, feelings related to sexuality remain, even in terminally ill patients, where the need for intimacy and closeness may be even greater than before the diagnosis [4].

In our study, opinions were divided about whether the advanced age of the patient constituted a barrier, which may reflect the variety of perceptions concerning the relationship between sexuality and older persons. This aspect has been found in international studies to be one of the most frequent factors [24] since older adults are usually associated with a lower interest in and concern with issues related to sexuality, even though many older people remain sexually active and value sexual intimacy as part of their quality of life [31].

It is noteworthy that the majority indicate that they have the necessary communication skills to talk about sexual health with their patients. However, approximately a third say that they are worried about making the patient uncomfortable by opening the topic and about a quarter of them report that they are afraid of offending the patient by doing so. This could reveal that the understanding that some practitioners have about ‘communication skills’ does not necessarily include the ability to hold back emotionally. This establishes one of the fundamental differences between an informative approach, centred around the problem, and an open discussion, centred around the particular needs of the patient.

Our study indicated no barrier related to the practitioner themself was considered as something that would prevent them from addressing sexuality, which constitutes a substantive difference with respect to other research, in which it is observed that the lack of knowledge, lack of training, discomfort in talking about sexuality and shame are considered fundamental barriers [19, 21, 28, 32]. The dismissal of the variables related to the practitioner may correspond to the fact that these types of barriers make assertions about their professional performance, which leads to more answers being given based on what is socially valued (social desirability), or that personal reluctance is not always completely conscious. On the other hand, organisational variables such as those already mentioned do not only hinder the clinical relationship, but also limit the opportunities for practitioners to have guided or personal spaces to reflect on, understand and work on their own relationship with areas such as sexuality, suffering, illness and death, which would make their own biases, prejudices and reluctance visible, allowing them to actively address the topic.

Despite the widespread recognition of the importance of sexuality in the quality of life, when asked specifically about their communication practices, less than two-thirds of the practitioners provided information about the possible side effects of cancer treatments on sexual health and only half say they report on the possible impact of oncological pathology on their sexuality. Even so, these percentages are high in relation to findings from international studies. Therefore, it could be concluded that those who voluntarily answered the survey have a greater interest in the topic and, therefore address it more frequently or see it as the expected response to show desired conduct in clinical practice. However, it is important to contrast providing information with asking about possible problems with sexuality, since the practitioners report asking only occasionally about their patients’ sexuality, which suggests that the information provided is given without being aware of the specific experiences and personal characteristics of the patients, which matches the biometric model and not patient-centred medicine [11].

The fact that the majority of respondents indicate that they only address patients’ sexuality when the anatomical location of the tumour affects the sexual organs, suggests that people with cancers located in other organs or haemato-oncological pathologies would not receive adequate information on this matter. This has a major influence on the inclusion of sexuality in clinical practice [33, 34]. This reinforces that the approach to sexuality tends to be focused on the relationship between cancer treatments and sexual dysfunction, conceptualising gender from genitality, which has been described as the ‘coital imperative’ [35]. This conceptualisation dismisses the variety of non-coital sexual practices, as well as the diversity of social and relationship context where the experience of sexuality and intimacy takes place [34].

Under the same hetero-normative paradigm, it can also be understood that one of the least common practices is investigating the sexual status of patients, despite the fact that sexual orientation was dismissed as a barrier. This suggests that there is no discriminatory behaviour *per se*, but rather there is an intention to provide equal care regardless of this information as a way to protect impartiality and objectivity. However, the literature suggests that the lack of knowledge and specific training on the sexual practices of sexual-affective atypicality and concern about inappropriate use of language lead to a resistance to opening up the topic of sexuality with LGBTIQA+ patients [34, 36]. This being the case, reservations about sexual status, and the limited understanding and knowledge of doctors about the specific needs of the group are linked to greater patient distress, negatively impacted emotional well-being, low levels of satisfaction with care and a loss of confidence in health services [37, 38].

## Limitations of the study

We must mention some limitations of the study. First, as far as we are aware, there are no validated scales in Chile for this topic, so we had to build one of our own based on other studies. Second, due to the voluntary nature of the studies that use on-line questionnaires, it is possible that there is a selection bias and, therefore, those who responded are those who were most interested in the topic. Thirdly, because it is a quantitative study, it is not possible to understand the phenomenon in its entirety. Therefore, future research could consider incorporating qualitative interviews to provide a more in-depth exploration of this topic. Finally, given that the study focused on the perspective of healthcare professionals, we do not know how cancer patients treated in Chile experience health care-related to sexual aspects.

## Conclusion

The results of this study show that the vast majority of oncologists in Chile believe that sexuality is an important part of the quality of life of their patients and recognise the impact that treatments and the disease itself have on sexuality. At the same time, they warn of the existence of organisational barriers that hinder adequate communication, as well as barriers related to the patient, but are not aware of their own barriers that prevent them from addressing the issue. Based on these results, we think that it is essential for healthcare professionals to be able to question their own clinical practice within a biomedical and hetero-normative paradigm that tends to determine which problems are fundamental, and which are of secondary importance. The backbone of the humanisation of oncology must be the active consideration of the integrality and complexity of the patients, taking into account their biography and not only their biology, so that the clinical practice is not understood through paternalistic terms, but based on the respect and promotion of the patient’s autonomy.

## Conflicts of interest

The authors have no conflicts of interest to declare. This study is based on DPMR’s thesis project for the Inter-University Master of Bioethics.

## Funding

This study did not have funding.

## Author contributions

DPMR was responsible for the study design, administering the questionnaire, analysing the results and writing the text. KG, in her role as thesis tutor, guided and supervised the work and gave a critical reading of the paper. CMV and SPS contributed to the critical analysis of the results and to the writing of the paper. All authors approved the paper submitted for publication.

## Ethical approval and consent to participate

The protocol was approved by the Scientific Ethics Committee of the Faculty of Medicine of the Clínica Alemana-Universidad del Desarrollo (2018-80), and each participant gave their informed consent before accessing the questionnaire.

## References

[1] Organización Mundial de la Salud (2022). Cáncer: datos y cifras. https://www.who.int/news-room/fact-sheets/detail/cancer.

[2] Zheng R, Wang S, Zhang S (2023). Global, regional, and national lifetime probabilities of developing cancer in 2020. Sci Bull (Beijing).

[3] Globocan and WHO (2021). Chile fact sheet. https://gco.iarc.fr/today/data/factsheets/populations/152-chile-fact-sheets.pdf.

[4] Mercadante S, Vitrano V, Catania V (2010). Sexual issues in early and late stage cancer: a review. Support Care Cancer.

[5] Jonsdottir JI, Zoëga S, Saevarsdottir T (2016). Changes in attitudes, practices and barriers among oncology health care professionals regarding sexual health care: outcomes from a 2-year educational intervention at a University Hospital. Eur J Oncol Nurs.

[6] Organización Mundial de la Salud (2018). La salud sexual y su relación con la salud reproductiva: un enfoque operativo.

[7] Vecino BS, Urbano SS, Trill MD (2003). Los trastornos sexuales. Psicooncologia.

[8] Saevarsdottir T, Fridriksdottir N, Gunnarsdottir S (2010). Quality of life and symptoms of anxiety and depression of patients receiving cancer chemotherapy longitudinal study. Cancer Nurs.

[9] Rose D, Ussher JM, Perz J (2017). Let’s talk about gay sex: gay and bisexual men’s sexual communication with healthcare professionals after prostate cancer. Eur J Cancer Care (Engl).

[10] Airaldi M (2010). Sexualidad y relaciones de pareja en mujeres mastectomizadas de una muestra paraguaya. Eureka.

[11] Hordern AJ, Street AF (2007). Communicating about patient sexuality and intimacy after cancer: mismatched expectations and unmet needs. Med J Aust.

[12] Flynn KE, Reese JB, Jeffery DD (2012). Patient experiences with communication about sex during and after treatment for cancer. Psychooncology.

[13] Tierney DK (2008). Sexuality: a quality-of-life issue for cancer survivors. Semin Oncol Nurs.

[14] Almont T, Bouhnik AD, Ben Charif A (2018). Sexual health problems and discussion in colorectal cancer patients 2 years after 1 diagnosis: a national cross-sectional study. J Sex Med.

[15] Albers LF, Belzen MA, Batenburg C (2020). Discussing sexuality in cancer care: towards personalized information for cancer patients and survivors. Support Care Cancer.

[16] Ussher JM, Perz J, Gilbert E (2013). Talking about sex after cancer: a discourse analytic study of health care professional accounts of sexual communication with patients. Psychol Health.

[17] Schover LR, Kaaij M, Dorst E (2014). Sexual dysfunction and infertility as late effects of cancer treatment. EJC Supplement.

[18] Hartmann U, Burkart M (2007). Erectile dysfunctions in patient-physician communication: optimized strategies for addressing sexual issues and the benefit of using a patient questionnaire. J Sex Med.

[19] Hautamäki K, Miettinen M, Kellokumpu-Lehtinen PL (2007). Opening communication with cancer patients about sexuality-related issues. Cancer Nurs.

[20] Dyer K, Nair R (2013). Why don’t healthcare professionals talk about sex? A systematic review of recent qualitative studies conducted in the United Kingdom. J Sex Med.

[21] Stead ML, Brown JM, Fallowfield L (2003). Lack of communication between healthcare professionals and women with ovarian cancer about sexual issues. Br J Cancer.

[22] De Araújo Ferreira SM, De Oliveira Gozzo T, Panobianco MS (2015). Barriers for the inclusion of sexuality in nursing care for women with gynecological and breast cancer: perspective of professionals. Rev Lat Am Enfermagem.

[23] Hernandez Sampieri R, Fernandez Collado C, Baptista Lucio P (2014). Definición del alcance de la investigación que se realizará: exploratorio, descriptivo, correlacional o explicativo. Metodología de la Investigación.

[24] Krouwel EM, Nicolai MPJ, Steijn-van Tol AQMJ (2015). Addressing changed sexual functioning in cancer patients: a cross-sectional survey among Dutch oncology nurses. Eur J Oncol Nurs.

[25] Aerny Perreten N, Domínguez-Berjón MAF, Astray Mochales J (2012). Tasas de respuesta a tres estudios de opinión realizados mediante cuestionarios en línea en el ámbito sanitario. Gac Sanit.

[26] Cunningham CT, Quan H, Hemmelgarn B (2015). Exploring physician specialist response rates to web-based surveys. BMC Med Res Methodol.

[27] Instituto Nacional de Estadística (2018). Compendio estadistico.

[28] Haboubi NHJ, Lincoln N (2003). Views of health professionals on discussing sexual issues with patients. Disabil Rehabil.

[29] Maree J, Fitch MI (2019). Holding conversations with cancer patients about sexuality: perspectives from Canadian and African healthcare professionals. Can Oncol Nurs J.

[30] Wazqar DY (2020). Sexual health care in cancer patients: a survey of healthcare providers’ knowledge, attitudes and barriers. J Clin Nurs.

[31] Gott M, Galena E, Hinchliff S (2004). “Opening a can of worms”: GP and practice nurse barriers to talking about sexual health in primary care. Fam Pract.

[32] Kotronoulas G, Papadopoulou C, Patiraki E (2009). Nurses’ knowledge, attitudes, and practices regarding provision of sexual health care in patients with cancer: critical review of the evidence. Support Care Cancer.

[33] Gilbert E, Ussher JM, Perz J (2011). Sexuality after gynaecological cancer: a review of the material, intrapsychic, and discursive aspects of treatment on women’s sexual-wellbeing. Maturitas.

[34] Perz J, Ussher JM, Gilbert E (2013). Constructions of sex and intimacy after cancer: Q methodology study of people with cancer, their partners, and health professionals. BMC Cancer.

[35] McPhillips K, Braun V, Gavey N (2001). Defining (hetero)sex: how imperative is the “coital imperative”. Womens Studies Int Forum.

[36] Stott DB (2013). The training needs of general practitioners in the exploration of sexual health matters and providing sexual healthcare to lesbian, gay and bisexual patients. Med Teach.

[37] Durso LE, Meyer IH (2013). Patterns and predictors of disclosure of sexual orientation to healthcare providers among lesbians, gay men, and bisexuals. Sex Res Soc Policy.

[38] Webster R, Drury-Smith H (2021). How can we meet the support needs of LGBT cancer patients in oncology? A systematic review. Radiography (Lond).

